# A Community-Engaged Approach to Developing a Mobile Cancer Prevention App: The mCPA Study Protocol

**DOI:** 10.2196/resprot.5290

**Published:** 2016-03-02

**Authors:** Selina Ann Smith, Mary Smith Whitehead, Joyce Sheats, Jeff Mastromonico, Wonsuk Yoo, Steven Scott Coughlin

**Affiliations:** ^1^ Institute of Public & Preventive Health Department of Family Medicine Medical College of Georgia Georgia Regents University Augusta, GA United States; ^2^ SISTAAH Talk Breast Cancer Support Group Miami, FL United States; ^3^ Educational & Collaborative Technology Center Georgia Regents University Augusta, GA United States; ^4^ Institute of Public & Preventive Health College of Dental Medicine Georgia Regents University Augusta, GA United States; ^5^ Department of Community Health and Sustainability Division of Public Health University of Massachusetts Lowell, MA United States

**Keywords:** smartphone applications, African Americans, breast cancer survivors, cancer prevention guidelines, dietary intake, physical activity

## Abstract

**Background:**

Rapid growth of mobile technologies has resulted in a proliferation of lifestyle-oriented mobile phone apps. However, most do not have a theoretical framework and few have been developed using a community-based participatory research approach. A community academic team will develop a theory-based, culturally tailored, mobile-enabled, Web-based app—the Mobile Cancer Prevention App (mCPA)—to promote adherence to dietary and physical activity guidelines.

**Objective:**

The aim of this study is to develop mCPA content with input from breast cancer survivors.

**Methods:**

Members of SISTAAH (Survivors Involving Supporters to Take Action in Advancing Health) Talk (N=12), treated for Stages I-IIIc breast cancer for less than 1 year, 75 years of age or younger, and English-speaking and writing, will be recruited to participate in the study. To develop the app content, breast cancer survivors will engage with researchers in videotaped and audiotaped sessions, including (1) didactic instructions with goals for, benefits of, and strategies to enhance dietary intake and physical activity, (2) guided discussions for setting individualized goals, monitoring progress, and providing or receiving feedback, (3) experiential nutrition education through cooking demonstrations, and (4) interactive physical activity focused on walking, yoga, and strength training. Qualitative (focus group discussions and key informant interviews) and quantitative (sensory evaluation) methods will be used to evaluate the participatory process and outcomes.

**Results:**

Investigators and participants anticipate development of an acceptable (frequency and duration of usage) feasible (structure, ease of use, features), and accessible mobile app available for intervention testing in early 2017.

**Conclusions:**

Depending on the availability of research funding, mCPA testing, which will be initiated in Miami, will be extended to Chicago, Houston, Philadelphia, and Los Angeles.

## Introduction

For women in the United States, breast cancer is the most common cancer and the second leading cause of cancer death [1]. Although white women have historically had higher incidence rates than African American (AA) women, the rates converged in 2012 [2]. Breast cancer death rates are higher for AA women compared to white women [3]. AA women are more likely to be diagnosed with breast cancer at younger ages and with more aggressive and advanced tumors. Although breast cancer survival in AA women has increased, survival rates remain lower than among white women [4].

Modifiable lifestyle risk factors related to energy balance may contribute to racial/ethnic disparities in breast cancer survival rates. Disparities in these factors are large and persistent, particularly between white and AA women. Recent data from the Behavioral Risk Factor Surveillance System revealed three disparity risk categories for AA women: (1) obesity (35.7% vs 23.7% for whites), (2) inadequate fruit and vegetable consumption (12.6% vs 17.4% for whites), and (3) low physical activity (63.8% vs 50.3% for whites) [5]. Lifestyle behavioral guidance to promote health and prevent disease is a cornerstone of American public health policy, yet relative to women of other races, AA breast cancer survivors are less likely to report adherence to the American Cancer Society [6] and the American Institute for Cancer Research (AICR) [7] cancer prevention guidelines.

Limited research on AAs exists because they have been underrepresented in studies examining health behaviors that improve breast cancer survival. One of the few studies with AA women, the Women’s Healthy Eating and Living Study, showed that at baseline, AA survivors were more likely to be obese (45% vs 25% for whites), to consume more calories from fat (+3.2%), to have fewer servings of fruits (-0.7/day), and were less successful at making and maintaining dietary changes than whites [8]. Greenlee et al [9] completed a randomized controlled trial with the commercially available Curves program, following 42 Hispanic and AA breast cancer survivors (BCSs) for 6 months. The trial resulted in weight loss that was not maintained at 6 months after the intervention. A community-based pilot study of 24 AA BCSs engaged in walking as physical activity (PA) [10] resulted in increases in steps walked per day and decreases in body mass index (BMI), body weight, and waist/hip circumferences, with most changes maintained at 3 months. A pre-post design, that included one of two weekly sessions dedicated to exercise, was used to test a 6-month intervention with 23 AA BCSs [11]. Participants experienced changes in weight, BMI, and social support. In a 16-week home-based motivational exercise program for 13 AA BCSs, there was a post-intervention increase in total minutes of PA and improved physical functioning [12].

To enhance our understanding of health behaviors that improve survival among AA BCSs, a lifestyle needs assessment of 240 members of the support group, SISTAAH (Survivors Involving Supporters to Take Action in Advancing Health) Talk, was completed. Most BCSs reported lack of adherence with cancer prevention recommendations for portion control (89%), body weight (68%), PA (83%), and intake of vegetables/fruits (75%), processed meat (54%), and red meat (53%) [13].

SISTAAH Talk members expressed interest in developing an alternative format for a lifestyle intervention. Rapid expansion of mobile technologies, including smartphone apps, provides such an opportunity. Currently, 85% of American adults own a mobile phone, and 45% own a smartphone, with usage higher among AAs (47%) relative to whites (42%) [14]. Half of all smartphone owners use their phones to search for health information, with 60% of all downloaded health-related apps involving weight loss and exercise [15].

A variety of apps relating to diet, nutrition, and weight control are available from platforms such as iPhone, Android, Nokia, and BlackBerry [16]. Traditional lifestyle interventions are facilitated and resource-intensive and may or may not be evidence-based. mHealth apps are mediated, less expensive, and rarely grounded in theory or evaluated using scientific methods [17-19]. To our knowledge, however, there are no apps that promote adherence to science-based cancer prevention guidelines. Adherence to these guidelines to prevent breast cancer recurrence is the primary focus of the Mobile Cancer Prevention App (mCPA) and such compliance may, in turn, contribute to improving health-related quality of life (eg, physical functioning, depression) [20]. Investigators postulate that employing principles of community-based participatory research (eg, unit of identity, building on resources within the community, cyclical and iterative processes) in developing a smartphone app, will result in widespread use and broader acceptance of cancer prevention guidelines. The purpose of this study is to partner with members of a breast cancer support group to develop a cancer prevention app for subsequent testing in a behavioral intervention study. Investigators hypothesize that a participatory process, one that engages individuals affected *by* the problem in developing solutions *to* the problem [21], will result in an acceptable (frequency and duration of usage), feasible (structure, ease of use, features), and accessible mobile cancer prevention app.

## Methods

### Study Population

Established in 1995 as the first breast cancer support group for women of color in South Florida, the goal of SISTAAH Talk is to provide a forum for African-Americans to communicate about and make sense of their diagnosis and treatment in order to achieve improved physical and mental health outcomes. SISTAAH Talk includes women from across Miami-Dade and Broward counties, reaching an average of 20 women each month through education, outreach, and research. In 2013, SISTAAH Talk breast cancer survivors (N=240) participated in focus group discussions and completed lifestyle assessments for the National Institute of Minority Health & Health Disparities-funded pilot study, Assessing Lifestyle Modification Needs & Experiences of African American Women [13]. Four focus group discussions (n=42; mean age 45.73 years, SD 7.91, range 35-75 years) identified barriers to and intervention approaches for enhancing dietary intake and PA. Themes emerging from content analysis converged into the following categories: practical tools (goal setting, self-monitoring), talk as central, led by BCSs, support group approach, “hands-on” or interactive nutrition (cooking) and physical activity education, and community-based (not community placed) research. The findings of these focus group discussions will be combined with theories in an intervention planning process between investigators and BCSs to inform the design of mCPA.

SISTAAH Talk members (n=12), treated for >1 year for Stages I-IIIc breast cancer, 75 years of age or younger, and English speaking/writing, were identified by leaders of the support group as good role models to participate in developing mCPA. Each applicant was interviewed by the principal investigator and support group facilitator to determine comfort level in participating in focus group discussions, key informant interviews, and app content development (eg, videotaping cooking demonstrations and PA).

### Participatory Engagement

SISTAAH Talk members participating in mCPA reviewed themes from focus group discussions during three telephone support group meetings with the principal investigator. To describe the participatory process of app development, Table 1 outlines contributions for each element used in the process.

**Table 1:** Participatory process of app development.

Element	Participant contribution	Investigator contribution	Outcome
Theoretical framework	Consider importance of lifestyle change to everyday life and survival	Identified theory to undergird BCSs’ belief that eating healthy and exercising regularly impacts survival	Health Belief Model; Theory of Planned Behavior
Peer-led activities	Include SISTAAH Talk members in all activities	BCSs as equal partners (facilitators, discussants) of cooking demonstrations, PA sessions, and focus group discussions	12 BCSs selected by SISTAAH Talk facilitator and mCPA principal investigator
Hands-on experiences	Mirror SISTAAH Talk activities by featuring BCSs cooking and exercising	Experiential nutrition and PA education	YouTube videos
Dietary intake	Feature recipes developed by AA community members; include practical, achievable recommendations that avoid drastic changes	Strategies and recipes promoting Cancer Prevention Guidelines	Down Home Healthy Living Cookbook
Physical activity	Walking, yoga, and strength training; advice from a BCS; consider different body sizes and practical, achievable goals	Symptoms such as lymphedema (swelling in an arm), arthralgia (pain in a joint), and neuropathy (numbness or weakness)	BCS-led walking, yoga, and strength training PA
Talk as central	The SISTAAH Talk approach to discussing lifestyle change	Incorporate beliefs, benefits, and barriers to lifestyle change as an instructional tool	Didactic sessions for goals, monitoring progress, and providing/receiving feedback

### Theoretical Framework

To provide a conceptual basis to undergird mCPA content, we will use the (1) health belief model (HBM) and (2) theory of planned behavior (TPB) (see Table 2). The HBM suggests that health-related cognitions for determining behavior considers the breast cancer survivor’s *belief* that lifestyle behaviors affect breast cancer recurrence, how *severe* the recurrence would be, and the *cost/benefits* of lifestyle change [22]. The TPB posits that health behavior is affected by past breast cancer experience and social norms (ie, lifestyle practices) more so than beliefs (ie, a link between breast cancer recurrence and lifestyle) [23].

**Table 2:** Theoretical framework by mCPA construct.

	Health Belief Model^a^			Theory of Planned Behavior^b^	
Component	HBM1	HBM2	HBM3	TPB4	TPB5
Education	×	×	×		
Instructions			×		
Goal setting					×
Social support				×	
Provide feedback					×
Prompt review					×
Self-monitoring		×			×
Teach use of cues		×			
Action planning				×	

^a^Health Belief Model: HBM1 (perceived costs); HBM2 (health benefits); HBM3 (cues for action).

^b^Theory of Planned Behavior: TPB4 (subjective norms/social support); TPB5 (behavior control).

### Cancer Prevention Guidelines and App Content

The proposed app will focus on the AICR guidelines for cancer survivors [7]: (1) Be as lean as possible without becoming underweight, (2) Be physically active for at least 30 minutes every day to help prevent cancer and prevent recurrence of cancer, (3) Avoid sugary drinks and limit consumption of energy-dense foods (particularly processed foods high in added sugar, low in fiber, or high in fat), (4) Eat more of a variety of vegetables, fruits, whole grains, and legumes such as beans, taking up at least 2/3 of your plate, (5) Limit consumption of red meats (such as beef, pork, and lamb) and avoid processed meats, taking up only 1/3 or less of the space on your plate, (6) If consumed at all, limit alcoholic drinks to two for men and one for women a day, and (7) Limit consumption of salty foods and foods processed with salt (sodium) by substituting herbs and spices high in phytochemicals (eg, basil, turmeric, paprika, thyme, and dill).

mCPA will include a flexible and autonomous approach to changing eating and PA behaviors. Tools on the app to support the development of self-regulatory skills and successful lifestyle change strategies will feature a range of instruments (eg, informational and self-monitoring) designed to enhance users’ awareness of and motivation to work toward cancer prevention guidelines. For example, informational tools will include options to view individual goals and plans and to access selected strategies (eg, eating out, planning meals, incorporating PA through the day). Self-monitoring tools will include options to receive personalized feedback on progress and access to support through links to social media (eg, Facebook, twitter). App users will establish dietary intake and PA goals, access strategies to support these goals, develop plans of how to meet them, review progress, and receive feedback on goal achievement. Employing a support group format, users will access cooking demonstrations, exercise instructions, and practical advice through links to YouTube videos.

A community-engaged process for transforming main dishes, side dishes, snacks, and desserts into healthier options and for presenting advice on dietary intake and physical activity for cancer prevention has been previously described [24]. Dishes from the cookbook with lifestyle tips, entitled, “Down Home Healthy Living (DHHL) 2.0,” [25] will be featured on the app. The purpose of the cookbook is to promote awareness of cancer prevention guidelines in African American communities. For the DHHL 2.0 cookbook with lifestyle tips, recipes were solicited from the National Black Leadership Initiative on Cancer community coalitions and dietary intake advice from participants in the Educational Program to Increase Colorectal Cancer Screening (EPICS). With guidance from a chef and registered dietitian, recipes were tested, assessed, and transformed. Lifestyle advice was obtained from focus groups. The cookbook has been distributed in print form to 2500 EPICS participants and shared electronically through 25 websites.

Physical activities for the app, specifically, walking, yoga, and strength training, were selected by SISTAAH Talk members during focus group discussions as options for inclusion in an intervention. A SISTAAH Talk member, BCS, and certified, licensed, insured fitness instructor will lead the PA experiential and instructional videos.

### Mobile Cancer Prevention App Development

A timeframe for developing the app is included in Table 3. The initial step in developing the app was establishment of a research protocol. SISTAAH Talk members met with investigators via telephone conference call August-September 2015 to complete the research protocol for obtaining Institution Review Board (IRB) approval, which was granted in October 2015. The Georgia Regents University IRB approved this research plan. Informed consent will be obtained from all participants.

During August-September 2015, content and format for the app was also outlined. SISTAAH Talk members will be videotaped during cooking and PA demonstrations at three time points (December 2015, January and February 2016). A prototype of the app will be developed, presented, revised, and tested during the final 6 months of the study (May-October 2016).

**Table 3:** App development timeline.

Steps	Oct. 2015	Dec. 2015	Jan. 2016	Feb. 2016	Mar. 2016	Apr. 2016	May 2016	June 2016	July 2016	Aug. 2016	Sept. 2016	Oct. 2016
Obtain IRB approval	✓											
Outline app content	✓											
Conduct focus groups		✓	✓	✓								
Tape cooking demos		✓	✓	✓								
Tape PA demos		✓	✓	✓								
Analyze focus group data					✓	✓	✓					
Conduct key informant interviews		✓	✓	✓								
Analyze key informant interview data					✓	✓	✓					
Develop app prototype								✓	✓			
Pretest draft app										✓		
Finalize app											✓	
Publish manuscripts				✓				✓				✓
Submit research proposals												✓

**Figure 1:**
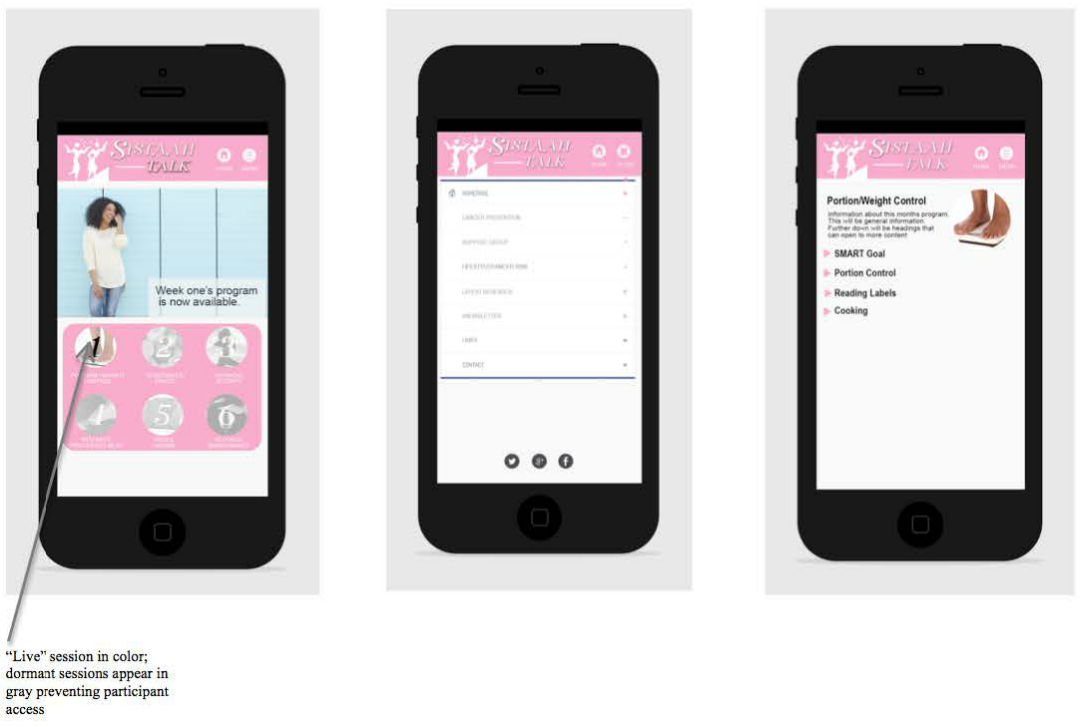
A sample of the mobile app interface before usability testing.

The proposed Web-based app will run like a Web page, with the app operating on an external server and the user accessing the app through the Web browser from a smartphone. The app will be developed and prototype-tested prior to going live on the smartphone. Participants will be provided a link to the app, which will transition from gray to color, with a pop-up message indicating that a component is now available for feasibility testing. Figure 1 provides a sample of the smartphone app interface.

Following informed consent, participants will engage in four activities to develop the content of the app. Each activity will be audio- and videotaped, with edited versions serving as content for mCPA. The first activity, didactic instructions, will provide information on goals for, benefits of, and strategies for enhancing dietary intake and PA. Researchers will present evidence linking lifestyle to breast cancer incidence and recurrence, review AICR guidelines for cancer survivors, and provide strategies for adhering to these guidelines, and address questions and concerns of the participants. Next, through interaction with investigators and other support group members, participants will develop app features for setting individual SMART (specific, measurable, actionable, realistic, time-sensitive) goals, monitoring progress, and providing/receiving feedback. Experiential nutrition education, with hands-on activities, including recipe development, modification, and taste testing through interactive cooking demonstrations, will be captured for the app. Finally, participants will engage in interactive PA, including walking at various levels (eg, power, speed interval, walking-to-jogging, and stretching), yoga (eg, physical postures, conscious breathing, and meditation), and strength training (eg, slowly progressive lifting of lightweight dumbbells) though a series of 30-minute sessions.

Focus group discussions and key informant interviews will be conducted to obtain participants’ feedback about app usefulness, identify the need for new system features and design requirements, and measure the acceptance of the mobile app and its features. Questions have been developed for the focus group discussions and key informant interviews based on the health belief model and theory of planned behavior (see Multimedia Appendix 1).

Four 90-minute focus groups with 12 SISTAAH Talk members and led by investigators, will explore three factors: performance expectancy, effort expectancy, and participant-centered factors. Performance expectance is defined as the degree to which a BCS believes that using the app will help attain lifestyle goals. Effort expectancy is the degree of ease associated with use of the app. Participant-centered factors include developing an app that is respectful of and responsive to user preferences, needs, and values, and ensuring that BCSs guide the development process. Acceptability will be determined by attitudes towards technology, anxiety, self-efficacy, and behavioral intention (eg, intent to use the app). Usability will be measured based on facilitating conditions and user-friendliness.

Three key informant interviews for each participant (n=36) will be conducted to ensure that perspectives across mCPA users are captured. Participants will be charged at baseline, midway, and postdevelopment with reviewing the app to make suggestions for adaptations and refinements and determine acceptability. Key informants will be provided access to the app, as it is being developed, and asked for feedback as part of a telephone follow-up.

### Data Collection

Tasting samples or prepared dishes specific to the cancer prevention guidelines (eg, replace refined grains with whole grains, reduce red and processed meat consumption, consume at least 5 fruits and vegetables per day, reduce portion sizes to lower body weight, and lose even small amounts of body weight) will be distributed. Participants will complete a sensory evaluation of the appearance, taste, texture, aroma, and overall acceptability of dishes prepared during cooking demonstrations. With a Likert scale, participants will be asked to rate each dish from 1 (unattractive; flavor did not appeal to me; inappropriate texture; unappetizing aroma; unacceptable) to 5 (extremely attractive; tasted great; great texture; smelled good; extremely acceptable). A discussion of the sensory evaluation results at the end of the cooking videos will be used to show app users the acceptability of the featured dishes.

Focus group discussions and responses to key informant interviews will be digitally recorded, transcribed verbatim, manually coded, and summarized. Data will be analyzed using Qualitative Content Analysis [26]. Coding steps will include developing preliminary themes creating additional codes based on themes that arise, developing non-substantive codes, and producing detailed codes for analysis of specific topics. NVivo 10 software for computer-assisted qualitative data analysis will be used to facilitate the coding process (ie, assessing the degree of agreement/disagreement across themes and calculating interrater reliability scores) [27]. A process of double coding (eg, 2 coders will code all items) will be utilized to calculate a level of agreement between coders and to determine consistency in coders. Recurring themes will be identified, the research team will come to a consensus on coded themes, and themes will be summarized for analysis.

After themes are applied, the first iteration of mCPA will be presented in a discussion forum. The discussion forum will include all SISTAAH Talk members attending monthly meetings, which will include the mCPA participants. All data related to developing the app and the app itself will be presented to determine acceptability (intention to use). Based on comments of the participants during the discussion, a second iteration of the app will be completed and made ready for testing. The second iteration of the app will be reviewed by the technology expert, principal investigator, and support group leaders, who will decide that the app is ready for pilot testing. This process will strengthen content validity and permit user input into intervention development.

### Data Analyses

mCPA acceptability, feasibility, and accessibility will be evaluated using descriptive statistics of frequency and proportion for discrete data and means and standard deviations for continuous data. All analyses will be accomplished with SAS version 9.4.

## Results

It is anticipated that development of an acceptable (frequency and duration of usage), feasible (structure, ease of use, features), and accessible mobile app will be available for intervention testing in early 2017.

## Discussion

### Principal Considerations

We expect the mobile-enabled, Web-based app (mCPA), which is theory-based and culturally tailored, to lead to multicenter, randomized controlled trials of its effectiveness in promoting healthy dietary intake and physical activity among African-American breast cancer survivors. An effective, research-tested mobile intervention to assist survivors in adopting and maintaining healthy behaviors would fill a gap in the current evidence based on culturally appropriate, low-cost interventions that can help African-American women maximize their health and quality of life following a breast cancer diagnosis. The availability of effective mHealth interventions for survivors would allow for future dissemination and implementation in order to reach large numbers of women at a relatively low cost.

Apps for patient self-management, such as the mCPA, empower women to take control of their own health and come to terms with what is often a frightening and anxiety-provoking diagnosis. The use of new technologies, apps, and social media for patient self-management of their illness and to reduce the risk of recurrence can be contrasted with computer-based programs that allow for providers to communicate with their patients as part of breast cancer treatment and survivorship care. The latter are closely tied to clinical care and may not be accessible or affordable to all groups of breast cancer survivors.

### Conclusions

Depending on the availability of research funding, mCPA testing, which will be initiated in Miami, will be extended to Chicago, Houston, Philadelphia, and Los Angeles. Results from the current study may lead to refinements, such as developing culturally tailored components of the app for ethnic subgroups of US blacks (eg, English-speaking Haitian-, and Caribbean-born blacks).

## Appendix

Multimedia Appendix 1 Topic and interview guides.

## Abbreviations

AA: African-American
AICR: American Institute on Cancer Research
BCSs: breast cancer survivors
BMI: body mass index
DHHL: Down Home Healthy Living
EPICS: Educational Program to Increase Colorectal Cancer Screening
HBM: Health Belief Model
IRB: Institutional Review Board
mCPA: mobile Cancer Prevention App
PA: physical activity
SISTAAH: Survivors Involving Supporters to Take Action in Advancing Health
SMART: specific, measurable, actionable, realistic, time-sensitive
TPB: Theory of Planned Behavior

## References

[1] American Cancer Society (2016). What are the key statistics about breast cancer?.

[2] DeSantis CE, Fedewa SA, Goding SA, Kramer JL, Smith RA, Jemal A (2016). Breast cancer statistics, 2015: Convergence of incidence rates between black and white women. CA Cancer J Clin.

[3] American Cancer Society (2014). Cancer Facts & Figures for African Americans 2013-2014.

[4] National Cancer Institute SEER Program (2015). Surveillance Epidemiology and End Results 2012.

[5] Centers for Disease Control and Prevention Prevalence Data.

[6] McCullough ML, Patel AV, Kushi LH, Patel R, Willett WC, Doyle C, Thun MJ, Gapstur SM (2011). Following cancer prevention guidelines reduces risk of cancer, cardiovascular disease, and all-cause mortality. Cancer Epidemiol Biomarkers Prev.

[7] American Institute for Cancer Research (2015). Our Cancer Prevention Recommendations.

[8] Paxton R, Jones L, Chang S, Hernandez M, Hajek RA, Flatt SW, Natarajan L, Pierce JP (2011). Was race a factor in the outcomes of the Women's Health Eating and Living Study?. Cancer.

[9] Greenlee HA, Crew KD, Mata JM, McKinley PS, Rundle AG, Zhang W, Liao Y, Tsai WY, Hershman DL (2013). A pilot randomized controlled trial of a commercial diet and exercise weight loss program in minority breast cancer survivors. Obesity (Silver Spring).

[10] Wilson D, Porter J, Parker G, Kilpatrick J (2005). Anthropometric changes using a walking intervention in African American breast cancer survivors: a pilot study. Prev Chronic Dis.

[11] Stolley M, Sharp L, Oh A, Schiffer L (2009). A weight loss intervention for African American breast cancer survivors, 2006. Prev Chronic Dis.

[12] Spector D, Deal AM, Amos KD, Yang H, Battaglini CL (2014). A pilot study of a home-based motivational exercise program for African American breast cancer survivors: clinical and quality-of-life outcomes. Integr Cancer Ther.

[13] Smith SA, Claridy MD, Whitehead MS, Sheats JQ, Yoo W, Alema-Mensah EA, Ansa BE, Coughlin SS (2015). Lifestyle Modification Experiences of African American Breast Cancer Survivors: A Needs Assessment. JMIR Cancer.

[14] Anderson M (2015). Technology Device Ownership: 2015.

[15] Health Fact Sheet (Tracking for Health).

[16] Kasbo A, McLaughlin R (2012). Mobile Health Applications: 2012 Study.

[17] Baker M (2011). Software for Shaping Up.

[18] Vollmer DD, Fair K, Hong YA, Beaudoin CE, Pulczinski J, Ory MG (2015). Apps seeking theories: results of a study on the use of health behavior change theories in cancer survivorship mobile apps. JMIR Mhealth Uhealth.

[19] Bender JL, Yue RYK, To MJ, Deacken L, Jadad AR (2013). A lot of action, but not in the right direction: systematic review and content analysis of smartphone applications for the prevention, detection, and management of cancer. J Med Internet Res.

[20] Touillaud M, Foucaut A, Berthouze SE, Reynes E, Kempf-Lépine A-S, Carretier J, Pérol D, Guillemaut S, Chabaud S, Bourne-Branchu V, Perrier L, Trédan O, Fervers B, Bachmann P (2013). Design of a randomised controlled trial of adapted physical activity during adjuvant treatment for localised breast cancer: the PASAPAS feasibility study. BMJ Open.

[21] Smith SA, Whitehead MS, Sheats JQ, Ansa BE, Coughlin SS, Blumenthal DS (2015). Community-based participatory research principles for the African American community. J Ga Public Health Assoc.

[22] Glanz K, Rimer B (2008). Health Behavior: Theory, Research, and Practice, 5th Edition.

[23] Ajzen I (1991). The theory of planned behavior. Organ Behav Hum Perf.

[24] Smith SA, Sheats JQ, Whitehead MS, Delmoor E, Britt T, Harris CL, Robinson-Flint J, Porche-Smith LM, Umeakunne KE, Coughlin SS (2015). Developing a Cookbook with Lifestyle Tips: A Community-Engaged Approach to Promoting Diet-Related Cancer Prevention Guidelines. Jacobs J Food Nutr.

[25] Down Home Healthy Living Cookbook 2.

[26] Schreier M (2012). Qualitative content analysis.

[27] Krefting L (1991). Rigor in qualitative research: the assessment of trustworthiness. Am J Occup Ther.

