# Supplementary material for: A Community-Engaged Approach to Developing a Mobile Cancer Prevention App: The mCPA Study Protocol
Source: JMIR Res Protoc. 2016 Mar 2;5(1):e34. doi: 10.2196/resprot.5290 (PMC4795322; doi:10.2196/resprot.5290)
Supplement: Multimedia Appendix 1 [file resprot_v5i1e34_app1.pdf]

Multimedia Appendix 1

FOCUS GROUP TOPIC GUIDE

<u>Focus Group #1</u>

What were the barriers to a) enhancing dietary intake; b) increasing physical activity?
How did you find working with the technology? Were there any technical difficulties?
How did you find the strategies included in the app to a) enhancing dietary intake; b)increase physical activity?
Did you have any additional strategies to help you reach your goals?

<u>Focus Group #2</u>

How would you describe the app to others?
How did you feel about letting people around you know that you were trying to a) enhance your dietary intake; b) increase your physical activity?
What was their reaction while you were a) enhancing dietary intake; b) increasing physical activity?
What were the key factors to keep you mindful about i a) enhancing dietary intake; b) increasing physical activity?

<u>Focus Group #3</u>

Were you comfortable using the app?
How often did you check the app during the day?
How did you react if your physical activity was lower than your goal?
What was the best thing about the app?
What did you like least about the app?
What improvements would you like to see to the app to improve its benefits?
How did you feel (in yourself) physically, mentally, and emotionally after using the app?
Would you using social media (Facebook, Twitter, etc.) to discuss your eating? exercising?
Do you see yourself continuing to use the app once the study period ends?
What would have made it easier or more motivating to keep it going?

KEY INFORMANT INTERVIEW GUIDE

For the sessions completed so far, please tell us about your experience:
Did you have problems downloading the app?
If yes, please tell us what problems you experienced?
How could the app be improved?
Please tell us any other comments that you have.
